# Oral Health Status of the Elderly at Tonga, West Region, Cameroon

**DOI:** 10.1155/2015/820416

**Published:** 2015-11-08

**Authors:** Yotat Michele Lolita, Agbor Ashu Michael, Ntumba Hubert, Djachechi Florence, Bolenge Jacques

**Affiliations:** ^1^Dental Department, Faculty of Medicine and Biomedical Sciences, Université des Montagnes, P.O. Box 206, Bangangté, Cameroon; ^2^Université de Kinshasa, Kinshasa, Democratic Republic of the Congo

## Abstract

*Objectives*. The aim of this study was to determine the oral health status of elderly persons in Tonga, West Region of Cameroon. *Methodology*. This is a cross-sectional study of persons of at least 65 years, living in Tonga village, West Region of Cameroon. *Results*. A total of 183 persons aged between 65 and 94 years, mean age of 73 years ±7 s.d., 83 (45,4%) males, and 100 (54,6%) females participated in the study. The most represented age range was 65–74 years (60.1%); 86 (47.3%) and elders above 65 constituted 1.8% of the total population. More than a third 117 (41.4%) had visible dental plaque, 117 (48,6%) had periodontal pockets >4 mm, 153 (54,1%) had teeth with total crown destruction, 70 (38.3%) had not lost a tooth, 23 (12.6%) had lost 1 tooth, 19 (10.4%) have lost at least 2 teeth, 100 (55.7%) were partially edentulous at the maxilla and 98 (53.6%) at the mandible, 2 (1.1%) were completely edentulous at the maxilla and 3 (1.6%) at the mandible, and 3.8% had removable dentures. The mean DMF index was 6.11 and 69.4% had dental caries. Risk factors to dental caries were toothbrushing and tobacco consumption while dental plaque was associated to pocket depth of 4–6 mm. Barriers to oral health care included ignorance 47 (25.7%), financial difficulties 124 (67.8%), and distance to the nearest clinic 12 (6.5%). * Conclusion.* The oral status of the elderly was generally poor.

## 1. Introduction

In many parts of the world, attaining old age is a rare privilege, though old age is often associated with some health challenges. Whether the added years at the end of the life cycle are healthy, enjoyable, and productive depends in part upon preventing and controlling a number of chronic diseases and conditions [1]. This is because around the age of 60 years chronic diseases associated with age increase and a slow and progressive deterioration of the oral health appears. Edentulousness, multiple restorations, root caries, attrition, periodontal diseases, and certain diseases of the oral mucosa are the main oral diseases of the elderly [2].

Therefore the elderly constitute a vulnerable population often suffering from various pathologies. Amongst them are oral pathologies; though considered negligible in terms of gravity, they constitute a nonnegligible factor of comorbidity [3]. These pathologies exert physiological, biological, and psychological changes associated with aging affecting their quality of life.

The world population is aging gradually. In Europe more than 17% of the population is more than 65 years old, compared to 12.4% in the United States [4]. In 2025, the global population has been previewed to be more than 830 million of people aged 65 years and above living mainly in developing countries [4]. As people age, their susceptibility to chronic and life-threatening diseases as well as acute infections increases, exacerbated by compromised immune systems. In addition to cancer, cardiovascular diseases, diabetes, and infections, poor oral health most notably tooth loss and severe periodontal conditions is prevalent in this age group [5]. Consequently population growth and longevity will project dental surgery in such a way that dental care for the elderly will be increased because of their increased oral health needs [4].

In Cameroon between 1976 and 1987, the number of elderly people had increased at an annual average rate of 2.1% whereas between 1987 and 2005 this subpopulation has an annual growth rate of 2.5%. At this rate, we expect even greater numbers of older people in the next ten years [6]. Cameroon is one of the sub-Saharan African countries where the population is rapidly increasing. Reports from the 3rd Cameroon household survey conducted in 2010 stated that Cameroon had approximately one million people above 60 years at January 2010 and predicted that this figure would increase to 1.1 million in 2015 and to 1.3 million in 2020 [6]. The results of the national population censuses and surveys have shown that, between 1950–1955 and 1990–2000, the decrease in mortality was spectacular, passing life expectancy of 36 years to 50 years or a life gain of 15 years in about 45 years, with an annual average gain of four months [6]. The number of people aged of at least 60 years increased from 441.450 in 1976 to 870.642 in 2005, with an average annual growth rate of 2.6% [6].

Most of the old people in Cameroon like in most developing countries enter old age after a life of poverty and deprivation, with poor access to healthcare, and inadequate diet in quality and quantity [7]. Majority of these elderly people suffer from various problems ranging from extreme poverty, ill-health, poor nutrition, lack of adequate care, shelter, clothing, isolation, and mental and physical abuse [7]. Many of them also suffer from age related health problems such as hypertension, diabetes, cancer, tuberculosis, arthritis, and ophthalmologic diseases especially poor eyesight [7, 8]. Factors associated with old age such as reduced salivary flow (in quality and quantity), lowered immunity, and the reduced ability of the body to repair may aggravate the process of the degradation of the oral tissues [7].

Oral diseases and tooth loss cause difficulties in chewing. This may in turn lead to selection of soft diets, which in most cases are composed of refined carbohydrates. This would easily predispose them to malnutrition and general ill health. Other difficulties associated with poor oral health status include difficulties in speech, deformed facial profile, and loss of self-esteem [7] which all together affect the quality of life of individuals.

Barriers to oral health care among the elderly are considerable. Impaired mobility, disabilities, poverty, inadequate financing for oral health care, and lack of oral health facilities impede access to care particularly for those who reside in rural areas.

In the recent years there has been a growing awareness in the dental profession that the dental needs of the elderly people have been generally neglected and that the problem requires attention.

In 1981, a collaboration working group of WHO/FDI formulated a number of oral health goals to be achieved as a measure of the attainment of “health for all by the year 2000.” As declared in 1979, one of these goals covered the elderly and stated that, by the year 2000, a 25% reduction in the present (1981) level of edentulousness at the age greater than 65 years will be achieved. It was also recommended that, greater than 50% of persons in this age group should be able to retain a minimum of 20 functional teeth [9]. However, this goal has not been attained yet.

Data on the oral health status and treatment needs of the elderly in majority of African countries and Cameroon in particular in a rural community are insufficient.

The aim of this study was to determine the oral health status of elderly persons in Tonga (a typical rural area), Nde Division of the West Region of Cameroon.

## 2. Methods

A cross-sectional survey was carried out for three months from May 1, to July 30, 2014, in Tonga, a subdivision of the Nde Division in the West Region of Cameroon (see Appendix  1 in Supplementary Material available online at http://dx.doi.org/10.1155/2015/820416) [10].

The Tonga subdivision is comprised of 3 major village groups: Badoumga (Banounga), Baloua, and Babitchoua with a surface area of 342 Km^2^ and a population of 10000 inhabitants. The headquarters of this subdivision are Tonga Town which is located 90 km from the Regional capital Bafoussam and 30 km from the divisional capital, Bangangte. Tonga is located along the Yaounde-Bafoussam road and is about 220 km from Yaounde the political capital of Cameroon.

The population is typically made up of 98% subsistent farmers producing food crops (rice cultivation, vegetables, and fruits) and cash crops like cocoa and coffee in small farms. It has a social welfare centre and 2 health centres (Tonga Catholic Health Centre, Maham Health Centre) and 1 medical doctor. There is no dental facility in this area and no oral health worker is known to have worked in this area. The climate is equatorially characterized by the alternation of two dry seasons and two rainy seasons.

### 2.1. Study Population

The study population consisted of the elderly of 65 years and above (as indicated by their age in their national identity card and medical records) who live in Tonga regardless of the gender.

#### 2.1.1. Sampling

The patients were selected using a stratified sampling technique as one of the 3 communities (Tonga village) was selected for the sampling. Subjects were now selected from house to house; therefore all individuals in each stratum who fulfilled the study criteria were invited to participate. The sample size was known at the end of the study as all houses were visited. An interviewer administered questionnaires to collect information from the subjects followed by a clinical oral examination carried out on the subject on a portable dental couch in a suitable place in the subject's resident.

#### 2.1.2. Inclusion Criteria

Inclusion criteria were only people of 65 years old and above, recently resident in Tonga permanently.

#### 2.1.3. Exclusion Criteria

Exclusion criteria were individuals who refused to give their consent.

#### 2.1.4. Data Collection

Data was collected using a structured open ended and closed ended questionnaire and a data captured sheet attached to the questionnaire for charting clinical examination results.

After obtaining administrative clearance, a door-to-door recruitment was done. Respondents were interviewed after reading and filling the consent form and their basic medical history obtained from the medical records and confirmed by an interview. Other pieces of information were obtained from a clinical examination out using examinations instruments under bright light. This included the examination of the oral hard and soft tissues and the presence and types of dental appliances in the oral cavity. Periodontal pockets were examined using a round ended graduated WHO periodontal probe.

The questionnaire was used for collecting information on the sociodemographic profile of the subjects, their general health status, their perception on their oral health, oral health seeking behaviour, mucosa, type of appliances used by the subject, and so forth.

Clinical examination was used in assessing possible risk factors and pathologies of oral pathologies using some indices as follows:

(i) The Silness and Loe's plaque index: to estimate the amount of soft and mineralized deposits on tooth surfaces, the scores were as follows:
   0 = no plaque;
   1 = presence of a thin visible layer of plaque by scraping the surface of the tooth with periodontal probe;
   2 = plaque deposits visible to the eye;
   3 = significant accumulation of plaque on tooth surfaces.
(ii) DMFT index (Decayed, Missing, and Filled Teeth): to determine the impact of dental caries on the teeth, we classified each tooth in one of the following categories:
   
*D* stands for tooth decay. It is used for any tooth which is observed to have visible recurrent caries and broken crown as a result of tooth decay or retained roots.
   
*M* stands for missing tooth for any tooth that is lost as a result of tooth extraction or for any other reason.
    
*F* stands for filled tooth as any tooth that has a permanent filling intact with no sign of caries lesion.
   
*N* is number of persons examined.
   DMF index = *D* + *M* + *F*/*N*+ [11].
(iii) The CPI (Community Periodontal Index): it was used to determine the periodontal status of the patients. It consists in dividing the mouth into six sextants and evaluating all teeth using a periodontal probe. Each sextant must have at least two functional teeth (i.e., teeth that are not extracted). Toothless sextants with just a functional tooth were considered absent and marked with “*X*” on the data captured sheet. The rating scale has five codes:
   0 = healthy;
   1 = spontaneous bleeding;
   2 = calculus;
   3 = pocket depth of 4–6 mm;
   4 = pocket depth > 6 mm [11].

### 2.2. Data Analysis

The data obtained were analysed using SPSS Version 17 software and Microsoft Excel 2007. The statistical test used was the Chi 2 with the significance level of *p* ≤ 0.05.

Ethical Clearance was obtained from the institutional research review board (Research and ethics committee) of the Université des Montagnes-Bangante. A research authorisation was obtained from the sub-divisional officer of Tonga and an informed consent from participants was also obtained (Appendices 2, 3, and 4).

## 3. Results

### 3.1. Demography

During our study the response rate was 97%; 183 persons aged between 65 and 94 years, with a mean age of 73 years ±7 s.d., 83 (45,4%) males, and 100 (54,6%) females participated in the study. The proportion of elderly people above 65 years old in Tonga was estimated to be 1.8% of the total population. Almost half 86 (47.3%) of the subjects were retired, 82 (45.1%) were farmers, 10 (5.46%) were house wife, and 5 (2.75%) are small traders.

### 3.2. The Age Range and Sex

The mean age was 77.5 years for women and 72.9 years for men. The most represented age range was 65–74 years (60.1%) (Table 1).

### 3.3. General Health Status

**Figure 1:**
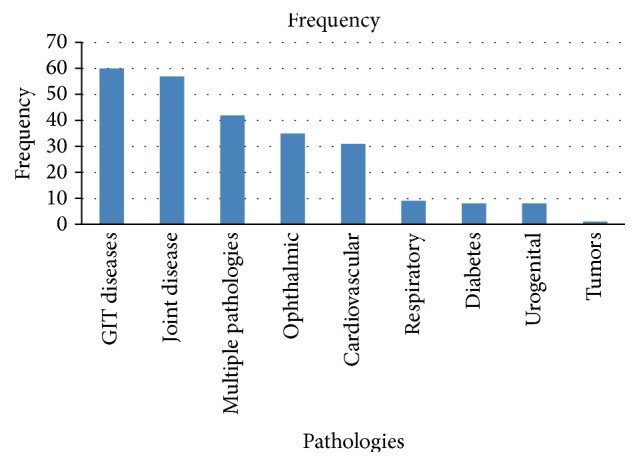
Distribution of the study population according to the general pathologies frequently encountered.

Digestive disorders 60 (33%) and joint disease 57 (31%), 42 (23%) multiple pathologies, and 35 (19%) ophthalmic problems were the most frequent (Figure 1).

### 3.4. Oral Hygiene Status

More than a third 69 (38.0%) of the respondents had poor oral hygiene, 55 (32.1%) had good oral hygiene, and 55 (30.1%) were fair.

**Figure 2:**
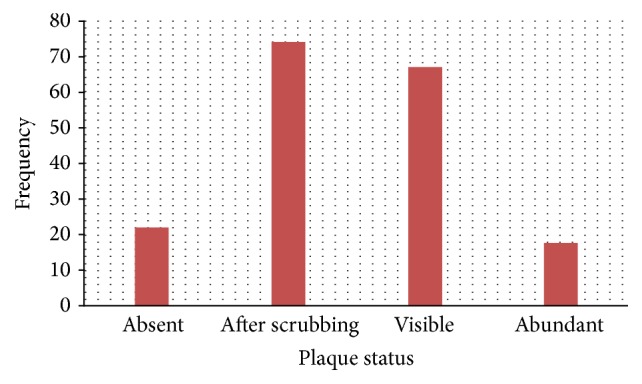
Distribution of population according to the amount of plaque present.

A third (36.5%) presented with plaque after scrabbling, the majority (41.4%) had visible dental plaque, and 12.7% had abundant plaque (Figure 2).

### 3.5. Oral Health Practice

#### 3.5.1. Toothbrushing Frequency

**Figure 3:**
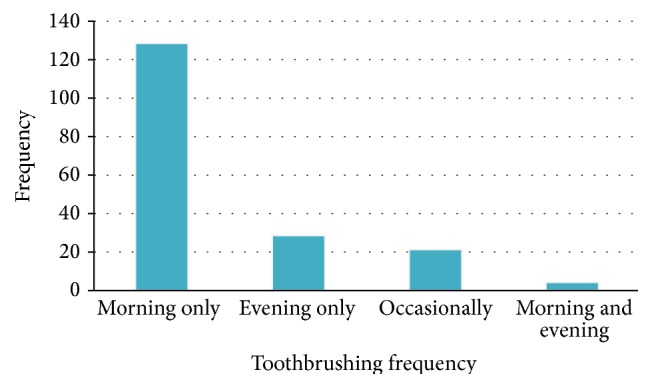
Distribution of the study population according to the toothbrushing frequency.

Three quarters 138 (75.4%) brush in the morning only, 36 (19.7%) had never brushed their mouth, and 9 (4.9%) brush in the morning and evening. All subjects brush before meals (Figure 3).

More than three quarters 143 (83.6%) of the subjects clean their mouth with a toothbrush, more than half 105 (57.4%) use toothpaste, almost half (42.6%) use the wood ash, 30 (16.4%) chew sticks, and only 1.6% use floss (Table 2).

### 3.6. Oral Pathologies

Three quarters 142 (78%) had gingivitis, 71 (39%) periodontitis, 56 (30%) dental caries, and 29 (15.8%) tooth wear (Table 3).

### 3.7. Periodontal Status

**Figure 4:**
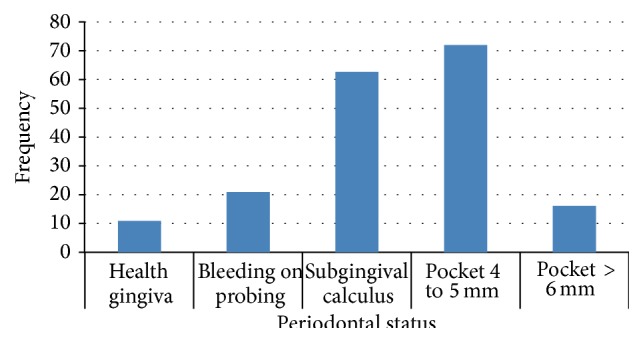
Distribution of the study population according to the periodontal status.

Periodontal pocket of 4–6 mm is the most represented (39.3%) dental pathology. Eighteen (10%) of the patients had mobile teeth (Figure 4).

A third 56 (30%) did not have any carious tooth, 27 (14%) had 1 missing tooth, and 26 (14%) had two missing teeth. More than a third 70 (38.3%) of the subjects had not lost a tooth, 23 (12.6%) have lost 1 tooth, 19 (10.4%) have lost at least 2 teeth, and 17 (9.3%) had more than 10 missing teeth (Table 4).

### 3.8. Filled Teeth

Only 1 (0.5%) patient had filled teeth.

### 3.9. DMFT Score

The majority had decayed teeth 127 (69, 4%), 112 (61, 7%) were with missing teeth, and 99 (54, 1%) were with teeth with total crown destruction.

The mean DMF index was 6.11.

### 3.10. Associated Risk Factors

The association between tooth decay and brushing only in the morning was statistically significant (*p* = 0,001). There was a strong association between tobacco consumption and tooth decay (*p* = 0,001) and also between visible dental plaque and pocket depth of 4–6 mm (*p* = 0,001) (Table 5).

### 3.11. The Type of Edentulousness

**Figure 5:**
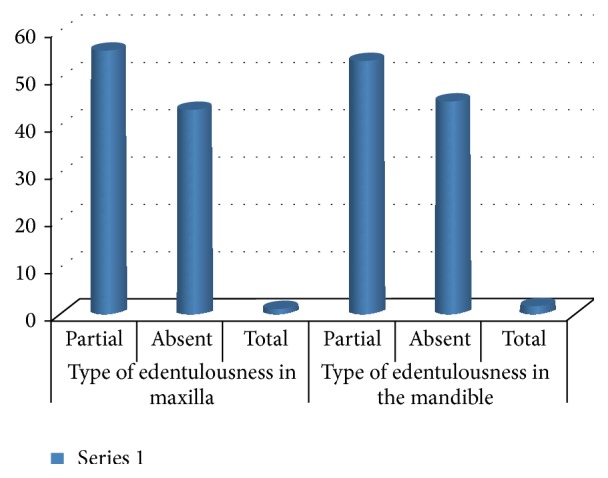
Distribution of the study population according to the type of edentulous.

More than half were partially edentulous, 100 (55.7%) at the maxilla and 98 (53.6%) at the mandible. A small proportion of the subjects were completely edentulous: 2 (1.1%) at the maxilla and 3 (1.6%) at the mandible (Figure 5).

**Figure 6:**
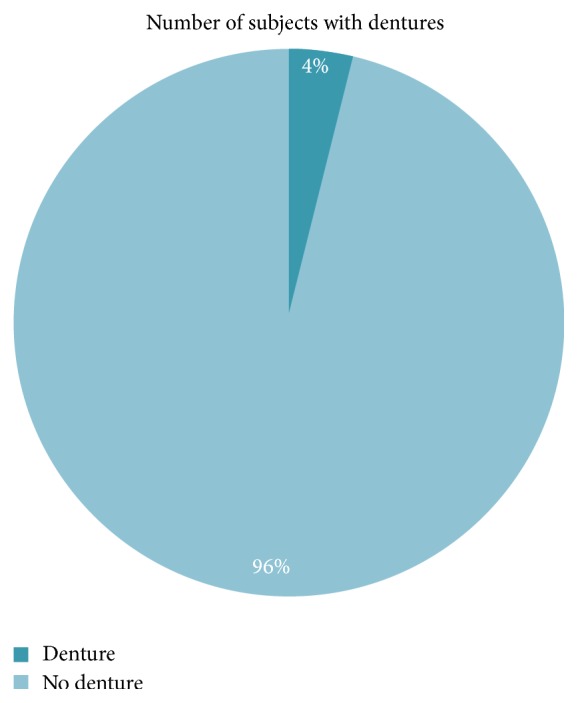
Distribution according to the wearing of dentures.

The majority (96.2%) of the edentulous subjects did not wear dentures (Figure 6).

### 3.12. Barriers to Access to Oral Health Care Facilities

The main barrier of access to oral health care facilities were financial 124 (67.8%), distance from clinic 12 (6.5%), and no reasons in particular 47 (25.7%) (Table 6).

## 4. Discussion

This study was carried out to assess the oral health status of the elderly in Tonga, a typical rural setting in Cameroon.

### 4.1. Sociodemographic Characters

This study showed that there was a female preponderance in the study group. These results support those of previous studies in which older women were predominantly represented [2]. The age group of 65–74 years is the most represented and the female was also dominant in this age groups. The reason for this female dominance is because most males are exposed to hazards during their active life and therefore die before old age. This is also reflected in the demographic pyramid of Cameroon where there are more males below the ages of 50 but more females above the ages of 55 [10].

The proportion of the elderly in the current study was estimated to be 1.8% of the entire study population; this is a deviation of the current demographic profile of the country that estimates the population of the elderly above 65 years to be 3.1% of the entire population [10]. The reasons for this could be that because of the improvement of the socioeconomic status of the country, many old people now reside in urban areas with their children where they are being catered for as culture demands that children should take care of the elderly; besides facilities for old people's home are nonexistent in the country. Old people stay with their children who are still procreating to cater for their grandchildren. It is not uncommon to find the elderly living with their children.

Most of the elderly were farmers and retired workers; this is because some Cameroon most civil servants who are still active, retire in the village because they can easily farm and live in a community which is less expensive and their immediate relations can cater for their immediate needs. The reasons for this are that the social security system of the country is not well developed to cater for the needs of the elderly [7].

### 4.2. General Health Status of Patients

The main systemic pathologies encountered amongst the elderly of our study were essentially digestive disorders, joint pains, ophthalmologic disorders, and cardiovascular diseases. This result is similar to that of Kanouté in Senegal who identified the main diseases encountered in these categories of persons, including heart disease, joint disease, and diabetes [12].

### 4.3. Oral Hygiene Status

Generally, the patients presented with poor oral hygiene and high calculus deposits. This is because in rural areas, dental services are completely absent and the distance to the nearest dental facility is very long. Besides, the culture of routine dental visits is not practiced in Cameroon. Most patients visit the clinic only when they have pain or an emergency dental problem [13].

### 4.4. Toothbrushing Frequency

Three quarters 138 (75.4%) brush in the morning only and 11.5% brush their teeth occasionally. This result is similar to Macqueronen in France which showed that 12% brushed their teeth occasionally [14]. He identified neglect as the main factor influencing the frequency of implementation of oral hygiene habits [14]. Apart from neglect, lack of oral health education or motivation was identified as the main cause of poor oral hygiene in our study.

### 4.5. Oral Hygiene Practices

The oral hygiene practices are far below standards. Apart from the use of tooth brushes and pastes in taking care of the oral hygiene, the elderly in the current study used materials like tooth picks, wood ashes and charcoal for their oral health care. These materials present with adverse effects on the hard and soft tissues of the teeth because of their abrasive nature. Chewing sticks is widely used for cleaning the teeth for religious [15] and socio-cultural reasons [15, 16] in the West and Central Africa countries. Norton and Addy (1989) compared the effectiveness of toothbrushes with chewing sticks on the oral hygiene and gingival health of adult Ghanaians revealed that plaque and gingivitis scores were higher in the chewing stick users, although these were primarily due to differences in men [16]. Men had poorer oral hygiene and gingival health than women, irrespective of the oral hygiene regimen. The same differences were apparent for city and rural dwellers, with no overall differences observed between these two groups. They concluded that the longer time that is necessary for cleaning with chewing sticks may explain the apparent reduced cleaning efficiency in men [16]. The antimicrobial substances contained in chewing sticks appear to provide no additional benefits to those produced by the antimicrobial activity of commercially available toothpastes [16]. This is contrary to another study carried in Nigeria which revealed that there was no significant difference on the effectiveness of toothbrushes and chewing sticks; after a six-week intervention period, postintervention readings were taken. They further noted that slight improvements were detected in the gingival status of those using the chewing sticks relative to those in the group using toothbrush [17]. The fact that, in the current study, visible plaque was found on the surface of the teeth of almost half of the participants means that their brushing frequency and technique need to be improved; another reasons for high plaque accumulation is because in rural areas, dental services are completely absent and the distance to the nearest dental facility is very long. The average income in most dependent elderly in rural areas is very small and very few of them can afford a toothbrush and toothpastes. Toothbrushing patterns using chewing sticks or twigs should be studied, standardised, and improved so that chewing sticks should be encouraged to be used in resource poor settings.

The culture of routine dental visits is not practiced in Cameroon. Most patients visit the clinic only when they have pain or an emergency dental problem [13]. This was also reported by Macqueron in France who showed that 34% of the elderly presented with visible dental plaque [14].

### 4.6. Periodontal Status

In the current study, the periodontal status was generally poor as the patients presented with high calculus deposits and more than a third presented with periodontal pockets of 4–6 mm. This result is similar to a study carried out in Saudi Arabia where calculus was the most prevalent (48.2%) finding among the subjects and was significantly more prevalent in men. Bleeding on probing and periodontal pockets was recorded in 24.1% and 19.3% of the subjects, respectively, with no statistically significant difference between men and women [18]. The frequency of periodontal pockets was similar to the findings of Kanouté (10.0%) [12] and Vatsana in Vietnam (9.8%) [19]. Inappropriate brushing technique and poor hygiene could explain the reasons for the deep periodontal pockets >4 mm.

### 4.7. Dental Status

The mean DMF in this study falls within the high range. This number is lower than the one found by Kanouté in Senegal [12] and Norway [20]. This could be as a result of the diet in rural areas which mostly are unrefined and fibrous.

The prevalence of tooth decay in our study was high. This result is similar to other studies carried out in France, Canada, South Africa, and Senegal [4, 10, 21, 22]. The level of education and access to oral health facilities of the population in these countries are different from that of Cameroon, which could explain why tooth decay is less common in Canada. Beside, most of these studies were carried in urban areas where health care facilities are easily accessible.

### 4.8. Missing Teeth, Teeth with Total Crown Destruction, and Filled Teeth

Tooth loss is always a result of tooth extraction and periodontal diseases. Severe periodontitis, which may cause tooth loss, is found in 5–15% of most populations [23]. The proportion of individuals with missing teeth in the current study is high as compared to another study carried out in France where the proportion of edentulousness was 26.9% [24]. This could be because of the high prevalence of periodontal diseases in rural areas. The current study demonstrated that 9.8% of subjects presented with mobile teeth which signifies that more teeth will be missing as the subjects grow older if intervention is not given. Lack of medical intervention is always neglected in rural communities in Cameroon because tooth loss and mobility is seen as a physiological process among the elderly. The high level of tooth mobility is because of ignorance about the causes of tooth loss and mobility as a result of periodontal diseases. It has also been observed that poor oral health status, together with a reduction of autonomy, can seriously affect the general health and increase the risk of death in elderly people [23].

More than half of the subjects in the present study presented with retained roots, a definite indication for tooth extractions. The fact that there are more people with missing teeth than people without missing teeth can be interpreted by the importance of the place of tooth extraction in our dental services. Indeed, in Cameroon, oral care is mainly dominated by tooth extractions [25].

The low rate of people with filled teeth demonstrates at a certain level, the low level of conservative treatment, delay diagnosis, and late presentation to the dental clinic at the stage where the tooth can no longer be restored which limits the patients to tooth extraction. This is a result of poor access to oral health care and inequalities in the distribution of the oral care workforce. It has been reported that, in some places in Cameroon, patients travel for at least 8 hours to get access to the nearest oral health care facility [25]; because of this, patients are obliged to visit traditional healers [13] or self-medicate [26].

### 4.9. The Type of Edentulousness and the Wearing of Dentures

The levels of edentulous at the maxilla and the mandible were very low as compared to other studies carried out in France and Kenya [7, 24]. This could be explained by the fact that the majority of persons in our study have several teeth indicated for extraction.

The wearing of dental prosthesis is one of the factors that can improve the quality of life of the subjects. In the current study a very low proportion of the subjects had dentures. This might correspond to the low level of edentulousness reported in this study. This is very low compared to that of Agbor et al. in France (65.9%) [27]. This is because dental prosthesis is expensive and not affordable by people in developing countries where the socioeconomic level is low; besides access to oral health care is limited and the rehabilitation prosthetic techniques are limited and insufficient.

### 4.10. Barriers to Access to Oral Health Care Facility

*Finances* and distance to the nearest health care facility are factors serving as barriers to access to oral health care facilities in most countries where there is inequality in oral health care delivery. This has been reported in Cameroon by Achembong et al. (2012) [25] and Agbor and Naidoo (2011) [13]. The nearest oral health care facility to Tonga is Bangangte which is 30 km away.

Some elderly people in the current study did not give any reasons for not attending a dental clinic. This might be because of ignorance, neglect, the utilisation of traditional and alternative medicine, and their nonbelief in orthodox medicine.

### 4.11. Associated/Common Risk Factors

Poor oral hygiene techniques and tobacco consumption where identified as risk factors for dental caries in the current study. This is similar to another study carried out in Fokoue a village in the west region of Cameroon where there was high caries rates amongst tobacco smokers. The subjects in the study also presented with poor oral hygiene [27].

The strong association between visible dental plaque and the periodontal pocket indicated that patients are not motivated and do not have access to basic oral health care. Therefore tobacco consumption and dental plaque can be noted as common risk factors in some dental problems and other systemic pathologies in the current study.

## 5. Conclusion

The elderly in Tonga presented with many systematic pathologies as well as poor oral health status: the oral pathologies commonly encountered are tooth decay, missing teeth, teeth with total crown destruction, and deep periodontal pockets of 4–6 mm. The level of oral hygiene is low with high calculus deposits. Oral hygiene practices are also below standards. The level of edentulousness was very low but very few subjects have prosthetic appliances. Barriers to access to oral health care facilities are distance, finance, and lack of interest in consulting a dentist. The major reasons for these dental problems are gross inequalities as a result of inadequate impact of the oral health care work force and facilities in rural areas in Cameroon and also no programme to take care of the oral health needs of the elderly.

*Weakness of the Study*. The oral health needs of the subjects and correlation of risk factors to specific diseases in the early was not also included. Only individuals resident in Tonga for at least 6 months were selected for the study.

## Recommendation

Oral health care policy making organization should advocate the inclusion and implementation of programmes that will facilitate the promotion of oral health care in rural areas in Cameroon.

The elder should be given free or low cost care during outreaches or in government facilities.

Studies should be conducted in other demographic settings (urban, semiurban, and rural areas) in Cameroon to evaluate the oral health status, treatment needs, and quality of life of the elderly in Cameroon.

The district health care community of Tonga health district should organise dental screening, oral health education, and training for the elderly at least once a year.

## Supplementary Material

Map of Tonga.

## Figures and Tables

**Table 1:** Distribution of the study population according to the age range and sex.

Age range	Men *N* (%)	Women *N* (%)	Total
[65–74]	61 (73,5%)	49 (79%)	110 (60,1%)
[75–84]	17 (20,5%)	44 (44%)	61 (33,1%)
[>85]	5 (6%)	7 (7%)	12 (3,3%)
Total	83 (45,4%)	100 (54,6%)	183 (100%)

**Table 2:** Distribution of the study population according to the oral hygiene practices.

Hygiene practice	Frequency (*n*)	Percentage (%)
Toothpick	180	98.4
Toothbrush	153	83.6
Toothpaste	105	57.4
Wood ash	78	42.6
Twig (chewing stick)	30	16.4
Floss	3	1.6

**Table 3:** Oral pathologies.

Pathology	Frequency	Percentage
Gingivitis	142	78%
periodontitis	71	39%
Dental caries	56	30%
Tooth wear	29	15.8%
Tooth mobility	18	10%
Oral mucosa lesions	16	9%
Oral cancer	2	1,1%

**Table 4:** Pathologies related to caries.

Carious teeth	Frequency	Percentage (%)	Missing teeth	Frequency	Percentage (%)
0	56	30,6	0	70	38,3
1	27	14,8	1	23	12,6
2	26	14,2	2	19	10,4
3	15	8,2	3	10	5,5
4	13	7,1	4	16	8,7
5	14	7,7	5	6	3,3
6	8	4,4	6	11	6,0
7	9	4,9	7	5	2,7
8	6	3,3	8	3	1,6
9	3	1,6	9	3	1,6
≥10	6	3.2%	≥10	17	9,3
**Total**	**183**	**100%**	**Total**	**183**	**100%**

**Table 5:** Carious pathology according to the associated factors.

Features	Caries	No caries	*p* value
Brushing only in the morning	92 (72.4)	37 (66)	0.001
Visible dental plaque	24 (18.9)	51 (91)	0.5
Tobacco	27 (21.3)	16 (28.6)	0.001
Alcohol	48 (37.8)	52 (92.8)	0.11

**Table 6:** Distribution of the study population according to the accessibility factors to oral care.

Barriers	Number (*N*)	Percentage (%)
Finance	124	67.8
Distance from clinic	12	6.5
None	47	25.7
Total	183	100
