# Supplementary material for: Oral Health Status of the Elderly at Tonga, West Region, Cameroon
Source: Int J Dent. 2015 Nov 8;2015:820416. doi: 10.1155/2015/820416 (PMC4655062; doi:10.1155/2015/820416)
Supplement: Supplementary file 1 — Map of Tonga. [file 820416.f1.pdf]

Appendices

Appendix 1. Location of Tonga

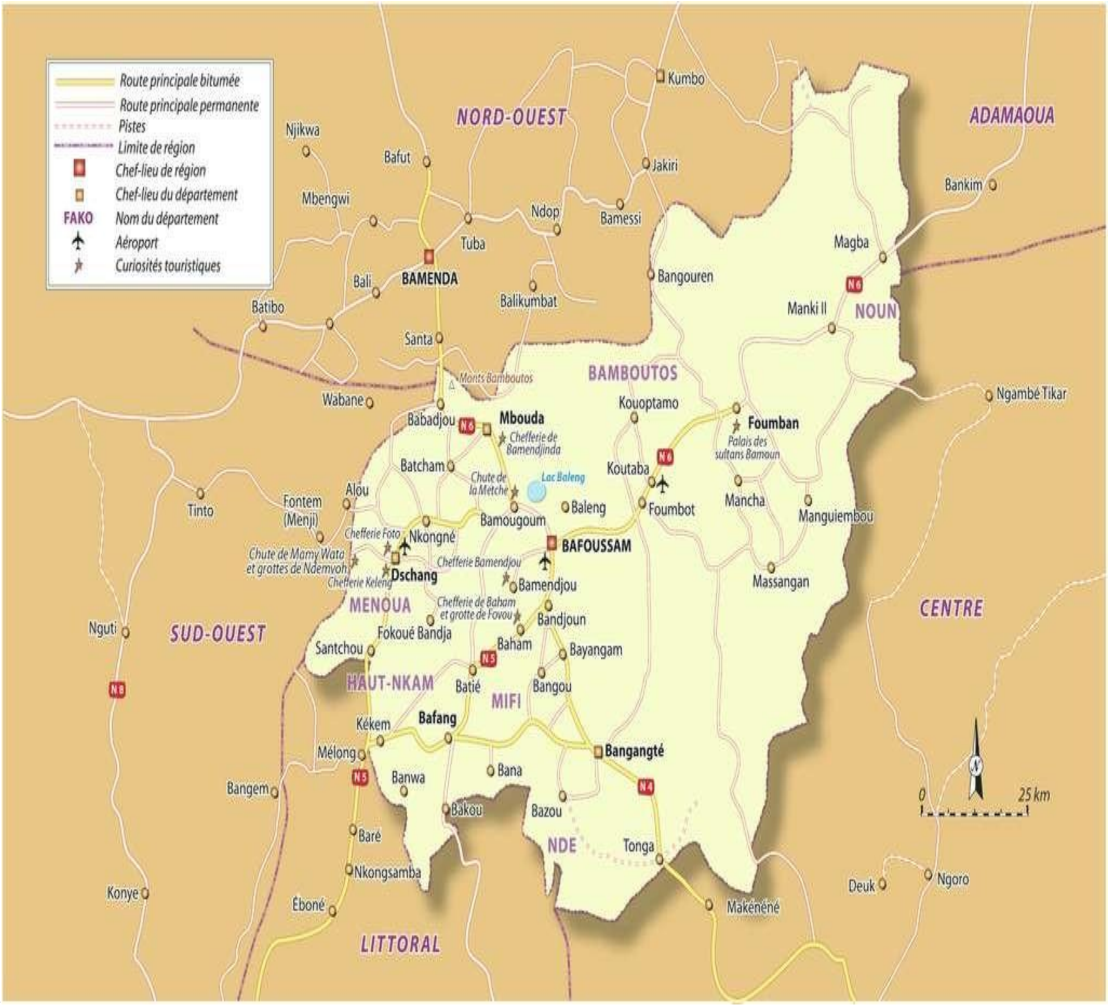

•

Appendix 2. Ethical clearance.

Université des Montagnes

Semper Altissimo Ascendere

COMITE INSTITUTIONNEL D'ETHIQUE

INSTITUTIONAL ETHIC COMMITTEE

Bangangté, le 30 JUIN 2014

AUTORISATION N° 2014/107/UdM/PR/CAB/CIE

CLAIRANCE ETHIQUE

Le Comité Institutionnel d'Ethique de l'Université des Montagnes (CIE-UdM) a examiné lors de sa session du 20 juin 2014, la demande de clairance éthique pour le projet de recherche intitulé **«Santé bucco-dentaire des personnes du troisième âge et plus dans la ville de Tonga»** soumis par **YOTAT Michele Lolita,** étudiante de Chirurgie Dentaire à l'Institut Supérieur des Sciences de la Santé de l'Université des Montagnes, Investigateur Principal.

Le sujet est digne d'intérêt et les objectifs de l'étude bien définis. La procédure proposée par l'Investigateur ne présente pas de risques préjudiciables aux participants. Par ailleurs, les considérations éthiques sont prises en compte. Le formulaire de consentement éclairé et la notice d'information des participants sont bien élaborés. Pour toutes ces raisons, le CIE-UdM autorise pour une période d'un an, la mise en œuvre du présent protocole.

Madame YOTAT Michele Lolita, Investigateur Principal, est tenue au respect scrupuleux du protocole approuvé et ne devra y apporter aucun amendement aussi mineur soit-il sans avis favorable du CIE-UdM. Elle est tenue de coopérer lors de toute descente du CIE-UdM pour le suivi de la mise en œuvre du protocole approuvé. Un exemplaire de la thèse/mémoire devra être remis au CIE-UdM, aux institutions d'accueil pour la recherche et aux autorités sanitaires du Cameroun.

La présente clairance peut être retirée en cas de non-respect de la réglementation en vigueur et des recommandations sus mentionnées.

En foi de quoi, la présente Clairance Ethique est délivrée pour servir et valoir ce que de droit.

Le Président du CIE-UdM,

Pr Lazare KAPTUE

BP 208 Bangangté, Cameroun T + (237)33 02 51 41 / 33 48 90 89 F + (237) 22 23 98 02 info@udesmontagnes.org www.udesmontagnes.org

Appendix 3 Questionnaires

SPECIFICATIONS

SECTION 1 : Identification

Date………………………..

Q101) Identification number ……………………

Q102) Location of the study………………………………………..

Q103) Gender………….   Q104) Age …………………………

Q105) Address………………   Q106) Profession   ……………………

SECTION2 : Medical and surgical history

Q201) Are you suffering from a pathology :

Cardiovascular   yes☐no☐do not know☐If yes, please specify…………

Hepatic   yes☐   no☐ do not know☐ If yes, please precise

Renal   yes☐   no☐   do not know☐ If yes, please precise …….

Endocrine   yes☐   no☐   do not know☐If yes, please precise ……

Neurological   yes☐   no☐   do not know☐ If yes, please precise …….

Infectious : VIH☐   Tuberculoses☐   Syphilis☐Hepatitis☐

Lungs :   yes☐   no☐   do not know☐

Q202) Are you on medication   :   yes☐   no☐If yes, which drugs ……

Q203) Toxic habits : cigarette☐   alcohol☐   drug☐

Q204)Hygiene practices: Do you use : - a toothbrush ? yes☐ no☐

- a twig? yes☐ no☐

- dental floss ? yes☐ no☐

- toothpick ? yes☐ no☐

- toothpaste ? yes☐ no☐

- coal ash ? yes☐ no☐

Q205) The frequency of toothbrushing : once a day☐2 times per day☐ occasionally☐

SECTION 3 : <u>Endo-buccal examination</u>

<u>Mucosal status</u>

Q301) Oral hygiene :

No dental plaque☐plaque after scrabbing☐ visible plaque☐abondant plaque☐

Q302) Attrition / erosion yes☐no☐

<u>Teeth status</u>

Q303)

0=healthy tooth

1=decay

2=filled and decay

3=filled without decay

C																
R																

(C=crown ; R= root)

4=missing

5=root debris

6=semi-impacted

T=trauma

7=not registered (C= crown ; R = root)

C																
R																

<u>Periodontal status</u>

Q304) Community Periodontal Index (CPI)

0= healthy tooth

1=bleeding

2=calculus

3= 4-5mm pocket depth

4=more than 6mm pocket depth

X= sextant ignored

Q305) gingival recession : yes☐ non☐

Q306) tooth mobility:degree I ☐degree II ☐degree III ☐

SECTION 4 : <u>Prosthetic Review</u>

Q401) Denture wearer : yes ☐ no ☐

Q402) Fractured denture : yes☐ no☐

Q403) Inadequate denture : yes☐ no☐

Q404) Do you know that dentures can be made ?

yes☐ no☐

Q405) Have you applied its making ? yes☐ no☐

Q406) If yes what was the answer ?……………………………..............

<u>Appendix 4</u> .<u>CONSENT FORM</u>

Title of the research project : Oral health of the elderly in Tonga.

PATIENTS INFORMATION SHEET

Principal Investigation : YOTAT Michele Lolita

N° approval of the Institutional ethics commitee 2014/107

Introduction of the research topic :The elderly constitute a vulnerable population, suffering from multiple pathologies. Amongst them the oral pathologies, eventhough they are not the most important in terms of gravity, act as a significant factor of comorbidity. We have to date found no qualitative research on the oral health of the elderly in Tonga.

Purpose : To determine the oral health status of the elderly in Tonga.

Procedure :If you agree the work is to answer a series of questions and do an oral examination.

Risk and potential discomfort : The time for filling the data sheets.

Confidentiality :Data collection sheets will carry a code for the anonymity of participants. The information gathered will be used only for science with medical confidentiality.

Participation : It is voluntary, however, knowing that refusal to participate or even withdrawal of the consent of the current study does not entail any harm to the participant.

Person to contact :

The principal investigator : YOTAT Michele Lolita, tel : 697034921

Co-director of the thesis :

Dr DJACHECHI Florence tel : 95969501

Dr. Agbor M A

Dr BOLENGE Jacques

Director of the thesis : Pr NTUMBA Hubert

B) CONSENT

I, the undersigned .... having read and understood the contents of this form and having received a copy, I voluntarily consent to the study by booking my right to waive whatever the time or reason.

date …………………….

signature of the participant
